# Menopause, postmenopausal hormone use and serum uric acid levels in US women – The Third National Health and Nutrition Examination Survey

**DOI:** 10.1186/ar2519

**Published:** 2008-09-26

**Authors:** A Elisabeth Hak, Hyon K Choi

**Affiliations:** 1 Departments of Immunology and Internal Medicine, Erasmus MC University Medical Center, Gravendijkwal 230, 3015 CE, Rotterdam, The Netherlands; 2 Rheumatology Division, Arthritis Research Centre of Canada, Department of Medicine, Vancouver General Hospital, University of British Columbia, 895 West 10th Avenue, Vancouver, BC V5Z 1L7, Canada

## Abstract

**Introduction:**

Despite the substantial prevalence of gout in the ageing female population, female hormonal influence has not been comprehensively examined. We evaluated and quantified the potential independent association between menopause, postmenopausal hormone use and serum uric acid levels in a nationally representative sample of women.

**Methods:**

Using data from 7662 women aged 20 years and older in the Third National Health and Nutrition Examination Survey (1988 to 1994), we examined the relation between menopause, postmenopausal hormone use and serum uric acid levels. We used multivariate linear regression to adjust for other risk factors for hyperuricaemia such as dietary factors, age, adiposity, alcohol use, renal function, hypertension and diuretic use.

**Results:**

Menopause was associated with higher serum uric acid levels. After adjusting for covariates, serum uric acid levels among women with natural menopause and surgical menopause were greater than premenopausal women by 0.34 mg/dl (95% confidence interval [CI], 0.19 to 0.49) and 0.36 mg/dl (95% CI, 0.14 to 0.57), respectively. Current postmenopausal hormone use was associated with a lower serum uric acid level among postmenopausal women (multivariate difference, 0.24 mg/dl [95% CI, 0.11 to 0.36]). The serum uric acid levels increased with increasing age categories (crude difference between 20 to 29 years and 70 years and over = 1.03 mg/dl, p for trend < 0.001), but this increase was not present after adjusting for other covariates (p for trend = 0.66).

**Conclusions:**

These findings from a nationally representative sample of US women indicate that menopause is independently associated with higher serum uric acid levels, whereas postmenopausal hormone use is associated with lower uric acid levels among postmenopausal women. The age-associated increase in serum uric acid levels in women may be explained by menopause and other age-related factors.

## Introduction

Despite the doubling of the incidence of gout among women over the past 20 years [1] and the substantial prevalence particularly in the ageing female population [2], little is known about the risk factors for gout and hyperuricaemia specifically among women. Given the important gender differences in the frequency of gout and serum uric acid levels, the risk factors for gout may vary between genders. A central factor behind these differences is thought to be female hormonal influence, but its magnitude has not been comprehensively examined and quantified. Thus, it is unknown if menopause is associated with serum uric acid levels independent of age and other covariates and if so, by what magnitude. Conversely, postmenopausal hormone use may be independently associated with lower serum uric acid levels, but no nationally representative information is available. Furthermore, previous studies reported an age-dependent increase in serum uric acid levels among women [3-5], but it is unknown if this increase is independent of menopausal effect or other age-related factors. To study these issues, we examined a nationally representative sample of women (the US Third National Health and Nutritional Examination Survey [NHANES III]) [6,7].

## Materials and methods

### Study population

Conducted between 1988 and 1994, the NHANES III included a representative sample of the non-institutionalised civilian US population, which was selected by using a multistage, stratified sampling design [6]. After a home interview, participants were invited to attend examination sessions where blood and urine specimens were obtained. For participants unable to attend the examination sessions for health reasons, a blood sample was obtained during the home interview. Our analysis was limited to women aged 20 years or older who attended the medical examination: of these women 7662 had complete information. We repeated our analyses among 7531 participants after excluding those who self-reported gout or were taking allopurinol or uricosuric agents (n = 131).

The NHANES III underwent institutional review board approval and written informed consent was obtained from participants.

### Uric acid measurement

Serum uric acid was measured by oxidisation with the specific enzyme uricase to form allantoin and hydrogen peroxide (Hitachi Model 737 Multichannel Analyzer, Boehringer Mannheim Diagnostics, Indianapolis, IN). Details about quality-control procedures have been published elsewhere [7]. Values are reported in milligrams per decilitre; to convert to micromoles per litre, multiply by 59.48.

### Assessment of menopausal status and postmenopausal hormone use

Participants were categorised as premenopausal (ovarian function intact), surgically menopausal (both ovaries removed surgically before cessation of menses) or naturally menopausal (nonsurgical loss of ovarian function) [8]. Participants with no history of reproductive surgery were classified as premenopausal if they reported having had a menstrual period during the previous 12 months and postmenopausal if they did not, consistent with World Health Organization criteria [8]. Women who had undergone a hysterectomy (without ovariectomy) that coincided with the date of the last menstrual period were assigned a menopausal classification on the basis of age (< 51 years were premenopausal; ≥ 51 years were naturally menopausal) [8]. Women with no history of hysterectomy or ovariectomy who were current users of hormone replacement therapy were classified in the same way. Women who had undergone bilateral ovariectomy that coincided with the date of the last menstrual period were classified as surgically menopausal [8]. Women who had undergone hysterectomy or ovariectomy after the date of the last menstrual period were classified as naturally menopausal. The amount of time since menopause was estimated as the difference in years between age at the time of the NHANES interview and self-reported age at the time of the last menstrual period or ovariectomy, whichever came first.

Women were classified as current users, past users or never users of postmenopausal hormone use on the basis of self-reported data from the examination questionnaire [8]. Duration of postmenopausal hormone use in years was also ascertained by self-report at the time of the examination.

### Assessment of covariates

The average daily intakes of total meat, seafood, dairy foods, sugar-sweetened soft drinks and coffee were derived from responses to a food frequency questionnaire. The food frequency questionnaire assessment of dietary intake has been shown to be a valid and reliable method of assessing average dietary consumption [9,10]. The NHANES III collected information on body measurements (including height and weight), medication use (including diuretics, anti-hypertensives, allopurinol and uricosuric agents), medical conditions (including self-reported physician-diagnosed diabetes, hypertension and gout) and serum creatinine levels. Glomerular filtration rate (GFR) was estimated by using the simplified Modification of Diet in Renal Disease study equation: GFR (ml/min/1.73 m^2^) = 186 × (serum creatinine level [mg/dl])^-1.154 ^× (age)^-0.203 ^× [0.742, if female] × [1.212, if black] [11-13]. Body mass index was calculated by dividing the weight in kilograms by the square of the height in metres.

### Statistical analysis

All statistical analyses were computed using survey commands of STATA (eg, SVYMEAN and SVYREG (StataCorp LP Texas)) to incorporate sample weights and adjust for clusters and strata of the complex sampling design. We used linear regression modelling to evaluate the relation between menopause, postmenopausal hormone use and serum uric acid levels. These models were adjusted for age; smoking status; body mass index; use of diuretics, beta-blockers, allopurinol and uricosuric agents; self-reported hypertension; GFR; and intake of total energy, total meats, seafood, dairy foods, sugar-sweetened soft drinks and coffee. When categorical analyses suggested linear trends across categories, statistical significance of trends were assessed in the final multivariate linear regression models using the median values of each category to minimise the influence of outliers.

We explored potential interactions by body mass index (< 25 kg/m^2 ^vs ≥ 25 kg/m^2^), hypertension (yes vs no) and alcohol use (abstainer vs drinker) by testing the significance of interaction terms added to our final multivariate models. For all difference estimates and odds ratios (OR), we calculated 95% confidence intervals (CI). All P values are two-sided.

## Results

The population's mean age was 46 years. The mean serum uric acid level was 4.64 mg/dl. The characteristics of the study population according to menopausal status are shown in Table 1. Postmenopausal women were older, more often hypertensive and more likely to use diuretics and uric acid-lowering medication than premenopausal women. These differences were larger when compared with natural menopause than surgical menopause. Postmenopausal women tended to consume less sweetened soft drinks, but more coffee than premenopausal women.

**Table 1:** Characteristics of women in the NHANES III according to menopausal status

**Variable**	**Premenopausal**	**Natural menopause**	**Surgical menopause**	**All participants**
Participants, n	4156	3047	459	7662
Age (years)	34	63	58	46
Body mass index (kg/m^2^)	26	27	27	26
Diuretic use (%)	2	18	15	8
Hypertension (%)	14	43	37	25
Alcohol intake (servings/day)	0.2	0.2	0.1	0.2
Total meat intake (servings/day)	1.0	0.9	0.9	1.0
Seafood intake (servings/day)	0.2	0.2	0.2	0.2
Dairy food intake (servings/day)	1.5	1.4	1.4	1.4
Sweetened soft drink intake (servings/day)	0.5	0.2	0.3	0.4
Coffee intake (servings/day)	0.9	1.3	1.1	1.0
Uric acid drug use^a ^(%)	0.0	1.3	0.5	0.5
Creatinine (mg/dl)	0.9	1.0	1.0	1.0

Data are presented incorporating sample weights and adjusted for clusters and strata of the complex sample design of NHANES III. ^a^Allopurinol and uricosuric agents.

Menopause was associated with a higher serum uric acid level. Unadjusted serum uric acid levels among women with natural menopause and surgical menopause were higher than among premenopausal women by 0.80 mg/dl (95% CI, 0.70 to 0.89) and 0.68 mg/dl (95% CI, 0.48 to 0.87), respectively (Table 2). After adjusting for age and other covariates, the differences were attenuated to 0.34 mg/dl in women with natural menopause and 0.36 mg/dl in women with surgical menopause, but remained significant (Table 2). When we excluded from the analysis participants who self-reported gout or were taking allopurinol or uricosuric agents (n = 131), the corresponding differences were 0.34 mg/dl in women with natural menopause and 0.37 mg/dl in women with surgical menopause (both p values ≤ 0.001). The independent association with menopause did not vary significantly among subgroups by body mass index (< 25 kg/m^2 ^vs ≥ 25 kg/m^2^), hypertension (yes vs no) and alcohol use (abstainer vs drinker) (p values for interaction > 0.3). Among women who had experienced natural menopause and had never used postmenopausal hormones, serum uric acid levels were higher in those who were younger than 40 years at menopause than in women who were 60 years or older at menopause (multivariate difference 0.50 mg/dl, [95% CI, 0.09 to 0.90]).

**Table 2:** Differences in serum uric acid levels (mg/dl) among women according to menopausal status^a^

**Menopausal status**	**Premenopausal**	**Natural menopause**	**Surgical menopause**
Participants, n	4156	3047	459
Unadjusted difference (95% CI)	0 (referent)	0.80 (0.70 to 0.89)	0.68 (0.48 to 0.87)
Age-adjusted difference (95% CI)	0 (referent)	0.43 (0.28 to 0.58)	0.37 (0.17 to 0.57)
Multivariate difference^b ^(95% CI)	0 (referent)	0.33 (0.18 to 0.48)	0.34 (0.12 to 0.55)
Multivariate difference^c ^(95% CI)	0 (referent)	0.34 (0.19 to 0.49)	0.36 (0.14 to 0.57)

^a^Uric acid levels are reported in milligrams per decilitre (mg/dl); to convert to μm/l, multiply by 59.48. Data are presented incorporating sample weights and adjusted for clusters and strata of the complex sample design of NHANES III. ^b^Adjusted for age, smoking status, body mass index, use of postmenopausal hormone, diuretics, beta-blockers, allopurinol and uricosuric agents, hypertension and glomerular filtration rate. ^c^Additionally adjusted for intake of alcohol, total meats, seafood, dairy foods, sugar-sweetened soft drinks, coffee and total energy. CI, confidence interval.

Among postmenopausal women, current users of postmenopausal hormones tended to be younger and less often hypertensive than past or never users of postmenopausal hormones (Table 3). Past users of postmenopausal hormones reported using urate-lowering medication less frequently.

**Table 3:** Characteristics of postmenopausal women in the NHANES III according to postmenopausal hormone use

**Variable**	**Never**	**Past use**	**Current**
Participants, n	2446	607	453
Age (years)	64	63	56
Body mass index (kg/m^2^)	28	27	26
Diuretic use (%)	18	18	16
Hypertension (%)	43	48	34
Alcohol intake (servings/day)	0.1	0.1	0.2
Total meat intake (servings/day)	0.9	0.9	0.9
Seafood intake (servings/day)	0.2	0.2	0.2
Dairy food intake (servings/day)	1.4	1.5	1.3
Sweetened soft drink intake (servings/day)	0.3	0.2	0.2
Coffee intake (servings/day)	1.2	1.2	1.3
Uric acid drug use^a ^(%)	1.4	0.4	1.4
Creatinine (mg/dl)	1.0	1.0	1.0

Data are presented incorporating sample weights and adjusted for clusters and strata of the complex sample design of NHANES III. ^a^Allopurinol and uricosuric agents.

Current postmenopausal hormone use was associated with a lower serum uric acid level among postmenopausal women. Unadjusted serum uric acid levels associated with current postmenopausal hormone use were lower than in women who had never used postmenopausal hormones by 0.44 mg/dl (95% CI, 0.30 to 0.58) (Table 4). After adjusting for age and other covariates, the difference was attenuated to 0.24 mg/dl, but remained significant (Table 4). When we excluded from our analysis participants who self-reported gout or were taking allopurinol or uricosuric agents (n = 117), the multivariate differences were 0.26 mg/dl (95% CI, 0.12 to 0.39) for current postmenopausal hormone use and 0.15 mg/dl (95% CI, 0.01 to 0.28) for past postmenopausal hormone use. The independent association with postmenopausal hormone use did not vary significantly among subgroups by body mass index (< 25 kg/m^2 ^vs ≥ 25 kg/m^2^), hypertension (yes vs no) and alcohol use (abstainer vs drinker) (p values for interaction > 0.06). Compared with no postmenopausal hormone use, the multivariate differences in serum uric acid levels were -0.38 mg/dl for duration of current postmenopausal hormone use of less than one year, -0.37 mg/dl for one to five years of use and -0.16 mg/dl (95% CI, -0.29 to -0.03) for more than five years of postmenopausal hormone use. The corresponding multivariate differences for duration of past postmenopausal hormone use were -0.14 mg/dl, 0.02 mg/dl and -0.28 mg/dl (95% CI, -0.52 to -0.04).

**Table 4:** Differences in serum uric acid levels (mg/dl) among postmenopausal women according to postmenopausal hormone use^a^

**Postmenopausal hormone use**	**Never**	**Past use**	**Current**
Participants, n	2464	607	453
Unadjusted difference (95% CI)	0 (referent)	-0.10 (-0.28 to 0.08)	-0.44 (-0.58 to -0.30)
Age-adjusted difference (95% CI)	0 (referent)	-0.09 (-0.26 to 0.09)	-0.34 (-0.49 to -0.18)
Multivariate difference^b ^(95% CI)	0 (referent)	-0.13 (-0.27 to 0.01)	-0.24 (-0.36 to -0.12)
Multivariate difference^c ^(95% CI)	0 (referent)	-0.13 (-0.27 to 0.02)	-0.24 (-0.36 to -0.11)

^a^Uric acid levels are reported in milligrams per decilitre (mg/dl); to convert to μm/l, multiply by 59.48. Data are presented incorporating sample weights and adjusted for clusters and strata of the complex sample design of NHANES III. ^b^Adjusted for age, sex, smoking status, body mass index, use of diuretics, beta-blockers, allopurinol and uricosuric agents, hypertension and glomerular filtration rate. ^c^Additionally adjusted for intake of alcohol, total meats, seafood, dairy foods, sugar-sweetened soft drinks, coffee and total energy. CI, confidence interval.

Serum uric acid levels did not vary significantly up to the age category of 40 to 49 years, but increased thereafter with increasing age categories (p for trend < 0.001) (Table 5). The unadjusted difference between 20 and 29 years of age and 70 years of age or older was 1.03 mg/dl. This increase in older age categories was attenuated after adjusting for menopausal status, but remained significant (p for trend < 0.001). However, when we additionally adjusted for GFR in the model, the association was no longer present (p for trend = 0.19). Instead of adjusting for GFR, when we additionally adjusted for creatinine levels, diuretic use and hypertension, the association was again not present (p for trend = 0.25). There was no significant trend in multivariate models adjusting for other covariates (p for trends > 0.53) (Table 5).

**Table 5:** Differences in serum uric acid levels (mg/dl) among women according to age categories^a^

**Age category (years)**	**20 to 29**	**30 to 39**	**40 to 49**	**50 to 59**	**60 to 69**	**≥ 70**	**P value for trend**
Participants, n	1627	1637	1203	904	987	1304	-
Unadjusted difference (95% CI)	0 (referent)	0.20 (-0.12 to 0.16)	0.12 (-0.04 to 0.28)	0.60 (0.44 to 0.75)	0.84 (0.69 to 1.00)	1.03 (0.90 to 1.16)	< 0.001
Menopause-adjusted difference (95% CI)	0 (referent)	0.00 (-0.14 to 0.14)	0.02 (-0.14 to 0.18)	0.27 (0.10 to 0.44)	0.45 (0.23 to 0.67)	0.62 (0.43 to 0.81)	< 0.001
Menopause-GFR Adjusted difference (95% CI)	0 (referent)	-0.09 (-0.25 to -0.08)	-0.13 (-0.35 to -0.10)	0.07 (-0.18 to 0.32)	0.17 (-0.18 to 0.52)	0.27 (-0.10 to 0.64)	0.19
Multivariate difference^b ^(95% CI)	0 (referent)	-0.23 (-0.37 to -0.08)	-0.33 (-0.53 to -0.14)	-0.22 (-0.44 to 0.00)	-0.16 (-0.46 to 0.14)	-0.06 (-0.39 to 0.27)	0.54
Multivariate difference^c ^(95% CI)	0 (referent)	-0.22 (-0.37 to -0.08)	-0.34 (-0.53 to -0.16)	-0.22 (-0.45 to 0.00)	-0.15 (-0.45 to 0.14)	-0.03 (-0.36 to 0.30)	0.66

^a^Uric acid levels are reported in milligrams per decilitre (mg/dl); to convert to μm/l, multiply by 59.48. Data are presented incorporating sample weights and adjusted for clusters and strata of the complex sample design of NHANES III. ^b^Adjusted for age, smoking status, body mass index, use of postmenopausal hormone, diuretics, beta-blockers, allopurinol and uricosuric agents, hypertension and glomerular filtration rate. ^c^Additionally adjusted for intake of alcohol, total meats, seafood, dairy foods, sugar-sweetened soft drinks, coffee and total energy. CI, confidence interval.

## Discussion

In this nationally representative sample of US women, we found that both natural and surgical menopause were associated with increased serum uric acid levels. The magnitude of associations was slightly larger than that associated with one daily serving of liquor (0.29 mg/dl), which was estimated based on NHANES III data [14]. In comparison, current postmenopausal hormone use was associated with lower uric acid levels among postmenopausal women. These associations were independent of other risk factors for hyperuricaemia such as age, body mass index, dietary risk factors, alcohol intake, renal function, hypertension and diuretic use. We also found a substantial increase in serum uric acid levels among women aged 50 years or older, but this increase was not present after adjusting for menopause and other age-related factors. These findings suggest that the increase was explained by menopause and other age-related factors that are associated with hyperuricaemia.

A biological mechanism that has been postulated to underlie the relation between menopause, postmenopausal hormone replacement use and serum uric acid levels is the impact of oestrogens on the renal tubular handling of uric acid [15-17]. Premenopausal levels of oestrogens in women may promote more efficient renal clearance of urate [15-17]. Serum urate concentrations in men average about 1 mg/dl higher than in women in adult life, but the serum uric acid levels in women increase substantially around the age of natural menopause, as shown in current and previous findings [3,4,15,18,19]. Furthermore, administration of oestrogen therapy to males was shown to decrease serum uric acid levels [17]. In parallel with our results, among women enrolled in the Heart and Estrogen-Progestin replacement Study, treatment with postmenopausal hormones resulted in a serum uric acid level of 0.2 mg/dl lower than placebo at one year of follow-up [20]. We observed no increasing hypouricaemic benefits with increasing duration of current postmenopausal hormone use of more than one year, although there were some increasing trends with past postmenopausal hormone use. Potential explanations for this include a survival effect, confounding of unmeasured covariates and a threshold effect of menopause on serum uric acid levels.

Age-related increases in serum uric acid levels among women have been reported by previous cross-sectional studies [3-5]. In contrast, serum urate levels did not vary significantly among men [3-5]. A study based on 3013 female residents of Tecumseh, MI [5] and a study based on 254 women in the UK [3] reported a rise in serum urate levels after age 50 to 54 years with a subsequent plateau. Another study based on 18,324 Japanese females reported increasing uric acid levels up to the age of 70 years or over [4]. Given the coinciding time periods, investigators inferred that this observation may be due to hormonal changes accompanying the menopause. Furthermore, previous case series found that the vast majority of female gout cases were diagnosed after menopause [15,18,19,21]. We found that serum uric acid levels among women increased from age 50 to 59 onwards and the increase extended up to the highest age category of 70 years of age and older. The increase attenuated substantially after adjusting for menopausal status, but remained significant, suggesting that menopause explains a substantial portion, but not all, of the age-associated increase among women. The remaining age-associated increase was explained by other age-related factors such as renal function, diuretic use and hypertension. Whether these factors also affect the risk of gout more strongly among women than among men remains to be examined in prospective studies with gout as the outcome.

Strengths and limitations of our study deserve comment. This study was performed in a nationally representative sample of US women; thus, the findings are likely to be generally applicable to US women. Although previous reports and biological plausibility suggest that female hormone use would affect the serum uric acid levels [15-17,20] as observed, a cross-sectional study design tends to leave uncertainty regarding the temporal sequence of exposure-outcome relations. Thus, confirming the relation between menopause, postmenopausal hormone use and incident hyperuricaemia or gout in a prospective longitudinal context would be valuable. Furthermore, it would be interesting to prospectively study if increasing serum uric acid trends associated with age translate into an increased risk of gout and, if so, if the trends can be explained by age-associated hyperuricaemic factors.

## Conclusion

In conclusion, our findings from a nationally representative sample of US women indicate that menopause is independently associated with higher serum uric acid levels, whereas postmenopausal hormone use is associated with lower uric acid levels among postmenopausal women. The age-dependent increase in serum uric acid levels in women may be explained by menopause and other age-associated factors.

## Abbreviations

CI: confidence intervals; GFR: glomerular filtration rate; NHANES III: the Third National Health and Nutritional Examination Survey; OR: odds ratios. 

## Competing interests

The authors declare that they have no competing interests.

## Authors' contributions

AEH and HKC contributed to the conception of the study, statistical analyses, interpretation of the results and preparation of the article.

## Acknowledgements

Dr. Choi holds the Mary Pack Arthritis Society of Canada Chair in Rheumatology. Dr. Hak is the recipient of an Erasmus MC Fellowship (Erasmus MC University Medical Center, Rotterdam, The Netherlands) and has been supported by the Foundation 'Vereniging Trustfonds Erasmus Universiteit Rotterdam', The Netherlands.
